# Crystal structure of 1,2-bis­(6-bromo-3,4-dihydro-2*H*-benz[*e*][1,3]oxazin-3-yl)ethane: a bromine-containing bis-benzoxazine

**DOI:** 10.1107/S2056989016016509

**Published:** 2016-10-25

**Authors:** Augusto Rivera, Jicli José’ Rojas, Jaime Ríos-Motta, Michael Bolte

**Affiliations:** a Universidad Nacional de Colombia, Sede Bogotá, Facultad de Ciencias, Departamento de Química, Cra 30 No. 45-03, Bogotá, Código Postal 110911, Colombia; b Institut für Anorganische Chemie, J. W. Goethe-Universität Frankfurt, Max-von Laue-Str., 7, 60438 Frankfurt/Main, Germany

**Keywords:** crystal structure, benzoxazines, phenolic resins, C—H⋯Br and C—H⋯O hydrogen bonds

## Abstract

The title benzoxazine molecule, C_18_H_18_Br_2_N_2_O_2_, was prepared by a Mannich-type reaction of 4-bromo­phenol with ethane-1,2-di­amine and formaldehyde. The title compound crystallizes in the monoclinic space group *C*2/*c* with a centre of inversion located at the mid-point of the C—C bond of the central CH_2_CH_2_ spacer. The oxazinic ring adopts a half-chair conformation. The structure is compared to those of other functionalized benzoxazines synthesized in our laboratory. In the crystal, weak C—H⋯Br and C—H⋯O hydrogen bonds stack the mol­ecules along the *b-*axis direction.

## Abstract 2

The solid-state structure of a 4-bromo­benzoxazine has been determined. The whole mol­ecule of the title compound is generated by inversion symmetry. This is a potential benzoxazine monomer for the preparation of phenolic materials.

## Chemical context   

In a continuation of our work on the synthesis and characterization of bis-1,3-benzoxazines, we have studied some of the chemical properties and determined the crystal structure of the title compound. Benzoxazines form an important class of benzo-fused heterocycles with a wide spectrum of biological activities. They are also emerging as desirable phenolic resin precursors because benzoxazines can undergo ring opening without emitting volatile materials during the curing process. This leads to a final cured product with excellent properties (Pilato, 2010 ▸). Normally, the incorporation of bromine can increase the flame-retarding properties and reduce the flammability of polymers (Li, *et al.*, 2010 ▸). Recently, we have investigated the crystal structures of analogous bifunctional benzoxazines namely 3,3′-(ethane-1,2-di­yl)bis­(6-substituted-3,4-di­hydro-2*H*-1,3-benzoxazine) (Rivera *et al.*, 2010 ▸, 2011 ▸, 2012*a* ▸,*b* ▸) that were prepared to investigate whether replacement of the substituents at the *para* position of the phenyl ring affects the electrophilic anomeric effect in the N–C–O sequence of the adjacent oxazine ring. In addition, as benzoxazine contains a tertiary nitro­gen atom, the lone-pair electrons may play an important role in the inter­action with guest mol­ecules but there are no reports on the inclusion properties of polybenzoxazines (Chirachanchai *et al.*, 2011 ▸). An X-ray structural study may therefore provide a better understanding of the ability of benzoxazines to act as novel host–guest compounds. In our opinion, the title compound also has potential applications in the production of new bromine-containing phenolic resins.

## Structural commentary   

**Figure 1:**
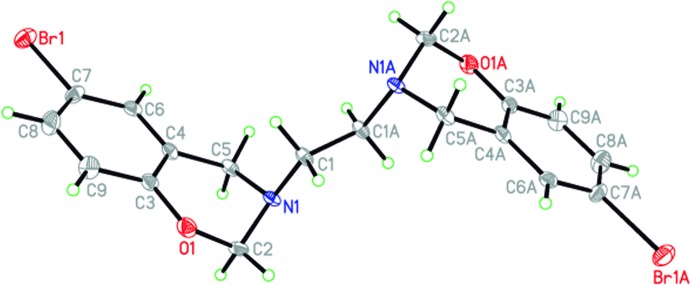
The mol­ecular structure of the title compound, Displacement ellipsoids are drawn at the 50% probability level. Atoms labelled with the suffix A are generated using the symmetry operator (1 − *x*, 1 − *y*, 1 − *z*).

The asymmetric unit of the title compound (Fig. 1 ▸), C_18_H_18_Br_2_N_2_O_2_, contains one half of the organic mol­ecule, an inversion centre generates the other half of the mol­ecule (symmetry operation: 1 − *x*, 1 − *y*, 1 − *z*). The six-membered oxazine heterocyclic ring adopts a half-chair conformation, with puckering parameters *Q* = 0.512 (2) Å, θ =129.6 (2)°, φ = 283.6 (3)°. This ring is analysed with respect to the plane formed by O1/C3/C4/C5, with deviations of the C2 and N1 atoms from this plane of 0.300 (6) and −0.320 (4) Å, respectively.
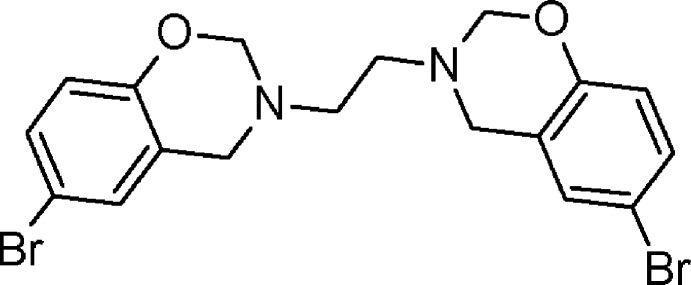


The C—C bond distances and angles of the aromatic rings were found to be normal. The C3—O1 bond length [1.372 (6) Å] is comparable with other previously reported C—O bond lengths for related structures [1.370 (10) and 1.388 (9) Å (Rivera *et al.*, 2012*a* ▸) and 1.376 (1) Å (Rivera *et al.*, 2011 ▸)], but is found to be shorter in the *p*-chloro derivative where C—O = 1.421 (2) Å (Rivera *et al.*, 2010 ▸). Inter­estingly, the C2—N1 and C2—O1 distances, 1.450 (5) and 1.456 (6) Å, respectively, are significantly different from the corresponding distances in the *p*-chloro derivative [1.369 (2) and 1.529 (2) Å, respectively; Rivera *et al.*, 2010 ▸]. Indeed, the values observed here are closer to those found in the analogous compound with no *p*-substituent on the aromatic ring (1.424 and 1.463 Å, respectively; Rivera *et al.*, 2012*a* ▸). This may indicate that the presence of the electron-withdrawing bromine atom does not significantly affect the adjacent oxazinic ring.

## Supra­molecular features   

**Figure 2:**
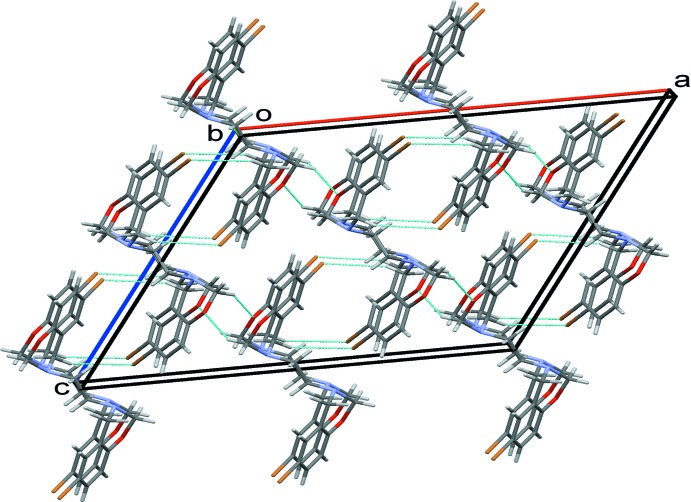
Packing diagram for the title compound, viewed along the *b* axis, with hydrogen bonds drawn as dashed lines.

In the crystal, weak C5—H5*B*⋯Br1 hydrogen bonds (Table 1 ▸) lead to the formation of inversion dimers with 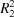
(12) ring motifs. These combine with the inversion symmetry of the mol­ecule to produce chains of mol­ecules along the *c* axis. Additional weak C2—H2*B*⋯O1 hydrogen bonds link these chains, stacking mol­ecules along the *b*-axis direction, Fig. 2 ▸.

## Database survey   

A database search yielded four comparable structures, namely 3,3′-(ethane-1,2-di­yl)bis­(6-methyl-3,4-di­hydro-2*H*-1,3-benzoxazine) (AXAKAM; Rivera *et al.*, 2011 ▸), 3,3′-ethyl­enebis(3,4-di­hydro-6-chloro-2*H*-1,3-benzoxazine), (NUQKAM; Rivera *et al.*, 2010 ▸), 3,3′-(ethane-1,2-di­yl)bis­(6-meth­oxy-3,4-di­hydro-2*H*-1,3-benzoxazine) monohydrate (QEDDOU; Rivera *et al.*, 2012*b* ▸), 3,3′-(ethane-1,2-di­yl)bis­(3,4-di­hydro-2*H*-1,3-benzoxazine) (SAGPUN; Rivera *et al.*, 2012*a* ▸). The Cl-substituted compound (NUQKAM) and the title compound are isomorphous. However, AXAKAM and SAGOUN have different space groups and in QEDDOU a solvent water mol­ecule is included in the crystal packing.

## Synthesis and crystallization   

An aqueous solution of formaldehyde (1.5 mL, 20 mmol) was added dropwise to a mixture of ethane-1,2-di­amine (0.34 ml, 5 mmol) and 4-bromo­phenol (1.73 g, 10 mmol) dissolved in dioxane (10 ml). The reaction mixture was stirred for 4 h at room temperature. Single crystals were obtained from this solution by slow evaporation of the solvent.

## Refinement   

Crystal data, data collection and structure refinement details are summarized in Table 2 ▸. All the H atoms were located in the difference electron-density map. C-bound H atoms were fixed geometrically (C—H = 0.95 or 0.99 Å) and refined using a riding-model approximation, with *U*_iso_(H) set to 1.2*U*_eq_ of the parent atom.

## Supplementary Material

Crystal structure: contains datablock(s) I. DOI: 10.1107/S2056989016016509/sj5510sup1.cif

Structure factors: contains datablock(s) I. DOI: 10.1107/S2056989016016509/sj5510Isup2.hkl

Supporting information file. DOI: 10.1107/S2056989016016509/sj5510Isup3.cml

CCDC reference: 1510085

Additional supporting information: crystallographic information; 3D view; checkCIF report

## Figures and Tables

**Table 1:** Hydrogen-bond geometry (Å, °)

*D*—H⋯*A*	*D*—H	H⋯*A*	*D*⋯*A*	*D*—H⋯*A*
C5—H5*B*⋯Br1^i^	0.99	3.04	3.951 (5)	154
C2—H2*B*⋯O1^ii^	0.99	2.64	3.506 (6)	146

Symmetry codes: (i) 
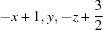
; (ii) 
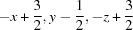
.

**Table 2:** Experimental details

Crystal data	
Chemical formula	C_18_H_18_Br_2_N_2_O_2_
*M*_r_	454.16
Crystal system, space group	Monoclinic, *C*2/*c*
Temperature (K)	173
*a*, *b*, *c* (Å)	19.464 (2), 5.9444 (7), 17.2225 (19)
β (°)	121.557 (7)
*V* (Å^3^)	1698.0 (3)
*Z*	4
Radiation type	Mo *K*α
μ (mm^−1^)	4.79
Crystal size (mm)	0.27 × 0.26 × 0.26

Data collection	
Diffractometer	STOE IPDS II two-circle
Absorption correction	Multi-scan (*X-AREA*; Stoe & Cie, 2001 ▸)
*T*_min_, *T*_max_	0.905, 1.000
No. of measured, independent and observed [*I* > 2σ(*I*)] reflections	3703, 1583, 1391
*R*_int_	0.078
(sin θ/λ)_max_ (Å^−1^)	0.607

Refinement	
*R*[*F* ^2^ > 2σ(*F* ^2^)], *wR*(*F* ^2^), *S*	0.056, 0.143, 1.07
No. of reflections	1583
No. of parameters	110
H-atom treatment	H-atom parameters constrained
Δρ_max_, Δρ_min_ (e Å^−3^)	1.40, −1.92

Computer programs: *X-AREA* (Stoe & Cie, 2001 ▸), *SHELXS* (Sheldrick, 2008 ▸), *SHELXL2014/7* (Sheldrick, 2015 ▸) and *XP* in *SHELXTL-Plus* (Sheldrick, 2008 ▸).

## supplementary crystallographic information

### Crystal data

**Table:**

C_18_H_18_Br_2_N_2_O_2_	*F*(000) = 904
*M**_r_* = 454.16	*D*_x_ = 1.777 Mg m^−^^3^
Monoclinic, *C*2/*c*	Mo *K*α radiation, λ = 0.71073 Å
*a* = 19.464 (2) Å	Cell parameters from 3703 reflections
*b* = 5.9444 (7) Å	θ = 3.6–26.0°
*c* = 17.2225 (19) Å	µ = 4.79 mm^−^^1^
β = 121.557 (7)°	*T* = 173 K
*V* = 1698.0 (3) Å^3^	Block, colourless
*Z* = 4	0.27 × 0.26 × 0.26 mm

### Data collection

**Table:**

STOE IPDS II two-circle diffractometer	1391 reflections with *I* > 2σ(*I*)
Radiation source: Genix 3D IµS microfocus X-ray source	*R*_int_ = 0.078
ω scans	θ_max_ = 25.6°, θ_min_ = 3.6°
Absorption correction: multi-scan (X-Area; Stoe & Cie, 2001)	*h* = −20→23
*T*_min_ = 0.905, *T*_max_ = 1.000	*k* = −7→7
3703 measured reflections	*l* = −20→20
1583 independent reflections	

### Refinement

**Table:**

Refinement on *F*^2^	Hydrogen site location: inferred from neighbouring sites
Least-squares matrix: full	H-atom parameters constrained
*R*[*F*^2^ > 2σ(*F*^2^)] = 0.056	*w* = 1/[σ^2^(*F*_o_^2^) + (0.099*P*)^2^] where *P* = (*F*_o_^2^ + 2*F*_c_^2^)/3
*wR*(*F*^2^) = 0.143	(Δ/σ)_max_ < 0.001
*S* = 1.07	Δρ_max_ = 1.40 e Å^−^^3^
1583 reflections	Δρ_min_ = −1.92 e Å^−^^3^
110 parameters	Extinction correction: *SHELXL-2014/7* (Sheldrick 2015), Fc^*^=kFc[1+0.001xFc^2^λ^3^/sin(2θ)]^-1/4^
0 restraints	Extinction coefficient: 0.0037 (8)

### Special details

**Table:**

Geometry. All esds (except the esd in the dihedral angle between two l.s. planes) are estimated using the full covariance matrix. The cell esds are taken into account individually in the estimation of esds in distances, angles and torsion angles; correlations between esds in cell parameters are only used when they are defined by crystal symmetry. An approximate (isotropic) treatment of cell esds is used for estimating esds involving l.s. planes.

### Fractional atomic coordinates and isotropic or equivalent isotropic displacement parameters (Å^2^)

**Table:**

	*x*	*y*	*z*	*U*_iso_*/*U*_eq_	
Br1	0.60406 (3)	−0.02845 (9)	0.93353 (3)	0.0226 (3)	
O1	0.67962 (18)	0.6369 (6)	0.7231 (2)	0.0176 (7)	
N1	0.6072 (2)	0.4166 (7)	0.5842 (2)	0.0130 (8)	
C1	0.5323 (2)	0.5473 (7)	0.5467 (3)	0.0141 (10)	
H1A	0.5430	0.7067	0.5401	0.017*	
H1B	0.5124	0.5406	0.5891	0.017*	
C2	0.6783 (3)	0.5494 (8)	0.6434 (3)	0.0155 (10)	
H2A	0.6811	0.6770	0.6082	0.019*	
H2B	0.7268	0.4552	0.6639	0.019*	
C3	0.6620 (3)	0.4788 (8)	0.7681 (3)	0.0141 (9)	
C4	0.6259 (2)	0.2721 (8)	0.7288 (3)	0.0142 (9)	
C5	0.6091 (2)	0.2158 (8)	0.6343 (3)	0.0140 (9)	
H5A	0.6513	0.1123	0.6400	0.017*	
H5B	0.5566	0.1372	0.5995	0.017*	
C6	0.6083 (2)	0.1218 (8)	0.7781 (3)	0.0147 (9)	
H6	0.5841	−0.0193	0.7526	0.018*	
C7	0.6265 (3)	0.1804 (8)	0.8655 (3)	0.0164 (9)	
C8	0.6619 (3)	0.3870 (9)	0.9044 (3)	0.0223 (10)	
H8	0.6741	0.4248	0.9639	0.027*	
C9	0.6787 (3)	0.5348 (8)	0.8552 (3)	0.0192 (11)	
H9	0.7021	0.6768	0.8807	0.023*	

### Atomic displacement parameters (Å^2^)

**Table:**

	*U*^11^	*U*^22^	*U*^33^	*U*^12^	*U*^13^	*U*^23^
Br1	0.0261 (4)	0.0229 (4)	0.0255 (4)	0.00128 (17)	0.0181 (3)	0.00527 (19)
O1	0.0161 (15)	0.0160 (18)	0.0166 (15)	−0.0043 (12)	0.0058 (13)	−0.0018 (13)
N1	0.0078 (16)	0.0135 (19)	0.0148 (17)	0.0005 (13)	0.0039 (14)	0.0003 (15)
C1	0.010 (2)	0.013 (2)	0.014 (2)	0.0037 (16)	0.0029 (19)	−0.0009 (18)
C2	0.012 (2)	0.016 (2)	0.019 (2)	−0.0051 (16)	0.0081 (18)	−0.0034 (18)
C3	0.0114 (19)	0.014 (2)	0.014 (2)	0.0000 (15)	0.0047 (17)	0.0021 (17)
C4	0.0093 (18)	0.015 (2)	0.014 (2)	0.0023 (16)	0.0030 (15)	0.0016 (17)
C5	0.0105 (19)	0.012 (2)	0.016 (2)	0.0009 (15)	0.0039 (16)	−0.0021 (17)
C6	0.0098 (18)	0.015 (2)	0.018 (2)	0.0015 (15)	0.0062 (16)	0.0022 (18)
C7	0.020 (2)	0.014 (2)	0.020 (2)	0.0032 (16)	0.0137 (18)	0.0057 (18)
C8	0.028 (2)	0.023 (3)	0.017 (2)	−0.001 (2)	0.0126 (19)	−0.0036 (19)
C9	0.023 (2)	0.015 (2)	0.019 (2)	−0.0003 (17)	0.011 (2)	−0.0020 (18)

### Geometric parameters (Å, º)

**Table:**

Br1—C7	1.909 (5)	C3—C4	1.402 (6)
O1—C3	1.372 (6)	C4—C6	1.393 (7)
O1—C2	1.455 (6)	C4—C5	1.519 (6)
N1—C2	1.450 (5)	C5—H5A	0.9900
N1—C5	1.462 (6)	C5—H5B	0.9900
N1—C1	1.470 (5)	C6—C7	1.397 (7)
C1—C1^i^	1.538 (8)	C6—H6	0.9500
C1—H1A	0.9900	C7—C8	1.396 (7)
C1—H1B	0.9900	C8—C9	1.373 (8)
C2—H2A	0.9900	C8—H8	0.9500
C2—H2B	0.9900	C9—H9	0.9500
C3—C9	1.398 (7)		
			
C3—O1—C2	113.7 (4)	C6—C4—C5	121.9 (4)
C2—N1—C5	108.0 (3)	C3—C4—C5	118.9 (4)
C2—N1—C1	112.6 (4)	N1—C5—C4	112.2 (4)
C5—N1—C1	113.8 (3)	N1—C5—H5A	109.2
N1—C1—C1^i^	110.2 (4)	C4—C5—H5A	109.2
N1—C1—H1A	109.6	N1—C5—H5B	109.2
C1^i^—C1—H1A	109.6	C4—C5—H5B	109.2
N1—C1—H1B	109.6	H5A—C5—H5B	107.9
C1^i^—C1—H1B	109.6	C4—C6—C7	119.5 (4)
H1A—C1—H1B	108.1	C4—C6—H6	120.3
N1—C2—O1	113.4 (4)	C7—C6—H6	120.3
N1—C2—H2A	108.9	C8—C7—C6	121.3 (4)
O1—C2—H2A	108.9	C8—C7—Br1	119.4 (4)
N1—C2—H2B	108.9	C6—C7—Br1	119.2 (4)
O1—C2—H2B	108.9	C9—C8—C7	118.9 (4)
H2A—C2—H2B	107.7	C9—C8—H8	120.5
O1—C3—C9	117.2 (4)	C7—C8—H8	120.5
O1—C3—C4	122.5 (4)	C8—C9—C3	120.8 (5)
C9—C3—C4	120.3 (5)	C8—C9—H9	119.6
C6—C4—C3	119.2 (4)	C3—C9—H9	119.6
			
C2—N1—C1—C1^i^	151.1 (5)	C1—N1—C5—C4	−77.1 (4)
C5—N1—C1—C1^i^	−85.6 (6)	C6—C4—C5—N1	161.5 (4)
C5—N1—C2—O1	−64.9 (5)	C3—C4—C5—N1	−20.1 (5)
C1—N1—C2—O1	61.6 (5)	C3—C4—C6—C7	0.3 (6)
C3—O1—C2—N1	47.8 (5)	C5—C4—C6—C7	178.7 (4)
C2—O1—C3—C9	166.5 (4)	C4—C6—C7—C8	0.1 (7)
C2—O1—C3—C4	−15.8 (5)	C4—C6—C7—Br1	−178.8 (3)
O1—C3—C4—C6	−178.7 (4)	C6—C7—C8—C9	0.2 (7)
C9—C3—C4—C6	−1.1 (6)	Br1—C7—C8—C9	179.2 (4)
O1—C3—C4—C5	2.9 (6)	C7—C8—C9—C3	−1.0 (7)
C9—C3—C4—C5	−179.5 (4)	O1—C3—C9—C8	179.2 (4)
C2—N1—C5—C4	48.7 (5)	C4—C3—C9—C8	1.4 (7)

Symmetry code: (i) −*x*+1, −*y*+1, −*z*+1.

### Hydrogen-bond geometry (Å, º)

**Table:**

*D*—H···*A*	*D*—H	H···*A*	*D*···*A*	*D*—H···*A*
C5—H5*B*···Br1^ii^	0.99	3.04	3.951 (5)	154
C2—H2*B*···O1^iii^	0.99	2.64	3.506 (6)	146

Symmetry codes: (ii) −*x*+1, *y*, −*z*+3/2; (iii) −*x*+3/2, *y*−1/2, −*z*+3/2.
